# Supplementary material for: Healthcare provider-to-patient perspectives on the uptake of teleconsultation services in the Nigerian healthcare system during the COVID-19 pandemic era
Source: PLOS Glob Public Health. 2022 Feb 9;2(2):e0000189. doi: 10.1371/journal.pgph.0000189 (PMC10021919; doi:10.1371/journal.pgph.0000189)
Supplement: S1 Survey — (DOCX) [file pgph.0000189.s001.docx]

**Online Survey: Teleconsultation services in the Nigerian healthcare system (Healthcare Provider)**

(Over the Phone/Video call medical consultations)

**Section 1: Informed Consent**

You are invited to participate in a web-based online survey on the acceptability and usefulness of teleconsultation services including medical consultations via Phone calls/text messages and Video calls in the Nigerian healthcare system.

Personal identifying information such as your name, email address, or IP address will NOT be collected. Therefore, your responses will remain anonymous. The information you provide will be treated with strict confidentiality and used solely for academic purposes.

**Contact:** iezeonwumelu@irsicaixa.es

**Please select your choice below. Clicking on the “Agree” button indicates that YOU have read the above information, YOU voluntarily agree to participate, and YOU are 18 years of age or older**


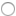
 Agree


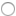
 Disagree

**Section 2: Demography**

1. **I have been a resident in Nigeria in the last 12 months**


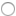
 Yes


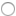
 No

1. **I am……**


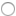
 Female


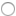
 Male


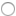
 Not listed


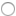
 Prefer not to say

1. **I am ......years old (Age)**


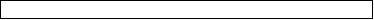


1. **What is the main focus of your clinical work?**


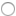
 General Practice


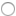
 Internal Medicine


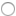
 OBS/GYN


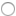
 Pediatrics


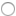
 Physiotherapy


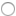
 Other (please specify)::
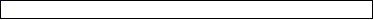


1. **Do you have any postgraduate qualifications**


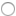
 Masters


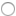
 PhD


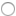
 Other

**Section 3: Healthcare background check**

1. **How frequently did you treat patients in a hospital/healthcare facility in the last 12 months?**


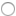
 Infrequently; at most 1 in the last 12 months


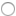
 Somewhat frequently; between 2 and 5 in the last 12 months


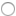
 Frequently; at least 1 per month


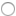
 Very frequently; at least 1 per week

1. **I have had more Hospital visitation cancellations or long-term rescheduling of appointments since the COVID-19 pandemic**


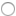
 Yes


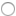
 No


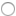
 Maybe

1. **Where do you usually deliver healthcare services**


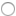
 Exclusively in the public health system


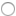
 Exclusively in private health settings


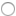
 In a combination of public health and non-public health settings


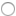
 Other

1. **Have you ever delivered healthcare/medical consultation to patients for any health condition over the phone and/or via video over the internet? (Please select all that apply)**


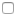
 No


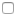
 Yes, over the phone.


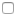
 Yes, via video over the internet (e.g. Whatsapp, Skype).

**Section 4: Telephone-delivered services**

(Medical consultations via phone calls/text messages)

1. **Using the phone to consult with a patient and prescribe medications would be easy for me.**


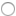
 Strongly agree
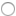
 Agree
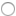
 Unsure
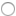
 Disagree
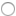
 Strongly disagree

1. **I would be as satisfied talking to a patient over the phone as I would be talking to a patient in-person in a consulting room at a healthcare facility.**


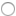
 Strongly agree
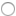
 Agree
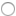
 Unsure
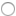
 Disagree
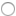
 Strongly disagree

1. **I like that there would be no physical contact with a patient when consulting over the phone.**


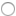
 Strongly agree
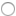
 Agree
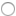
 Unsure
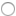
 Disagree
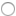
 Strongly disagree

1. **Delivering medical consultations and treatment to a patient over the phone would be a convenient form of healthcare for patients.**


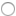
 Strongly agree
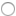
 Agree
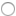
 Unsure
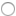
 Disagree
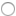
 Strongly disagree

1. **Delivering medical consultations and treatment to a patient over the phone would save the patient time.**


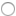
 Strongly agree
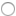
 Agree
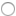
 Unsure
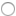
 Disagree
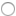
 Strongly disagree

1. **A session of medical consultations over the phone should cost patients...**


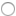
 50% more than the cost of a face-to-face hospital visitations


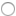
 25% more than the cost of a face-to-face hospital visitations


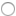
 The same cost as a face-to-face hospital visitations


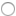
 25% less than the cost of a face-to-face hospital visitations


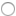
 50% less than the cost of a face-to-face hospital visitations

1. **I would be interested in being involved in a service offering medical consultations over the phone for patients.**


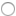
 Strongly agree
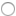
 Agree
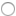
 Unsure
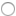
 Disagree
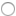
 Strongly disagree

1. **Video over the internet (Whatsapp, Skype, Zoom) will improve my teleconsultation experience and satisfaction**


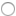
 Strongly agree
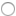
 Agree
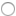
 Unsure
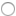
 Disagree
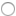
 Strongly disagree

**Section 5: Telephone-delivered services during the COVID-19 pandemic physical distancing and movement restrictions**

1. **Using the phone would be a useful (practical) and effective way for patients to receive healthcare service from a Doctor/Health professional during COVID-19 pandemic**


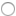
 Strongly agree
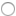
 Agree
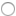
 Unsure
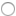
 Disagree
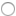
 Strongly disagree

1. **Using the phone would be an affordable way for patients to receive healthcare services during COVID-19 pandemic**


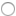
 Strongly agree
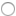
 Agree
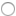
 Unsure
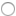
 Disagree
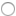
 Strongly disagree

1. **Using the phone would be a safe way for patients to receive healthcare services during COVID-19 pandemic**


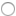
 Strongly agree
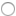
 Agree
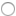
 Unsure
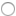
 Disagree
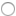
 Strongly disagree

1. **I will prefer to use Telephone delivered medical consultations ONLY during pandemics such as COVID-19**


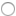
 Strongly agree
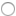
 Agree
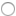
 Unsure
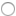
 Disagree
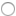
 Strongly disagree
