# Supplementary material for: Healthcare provider-to-patient perspectives on the uptake of teleconsultation services in the Nigerian healthcare system during the COVID-19 pandemic era
Source: PLOS Glob Public Health. 2022 Feb 9;2(2):e0000189. doi: 10.1371/journal.pgph.0000189 (PMC10021919; doi:10.1371/journal.pgph.0000189)
Supplement: S1 Table — (DOCX) [file pgph.0000189.s004.docx]

**S1 Table: Results-Association between Healthcare Providers Demographic Characteristics and Perceptions on Telemedicine Use**

|  | Agreed that they liked  that there would be no physical contact with patients when consulting over the phone | Disagreed that they liked  that there would be no physical contact with patients when consulting over the phone | P-Value |
| --- | --- | --- | --- |
| **Age** |  |  | 0.197 |
| 18-24 years | 1(0.6) | 15 (9.5) |  |
| 25-48 years | 33 (20.9) | 109 (69.0) |  |
| **Geopolitical Zone** |  |  | 0.075 |
| North East | 2 (1.3) | 3 (1.9) |  |
| North-West | 0 (0.0) | 5 (3.2) |  |
| North Central | 3 (1.9) | 4 (2.5) |  |
| South-West | 8 (5.1) | 12 (7.6) |  |
| South-East | 10 (6.3) | 44 (27.8) |  |
| South-south | 11 (7.0) | 56 (35.4) |  |
| **Gender** |  |  | 0.235 |
| Female | 17 (10.8) | 46 (29.1) |  |
| Male | 17 (10.8) | 78 (49.4) |  |
| **Recent Professional Development Status** |  |  | 0.826 |
| Consultancy | 1 (0.6) | 3 (1.9) |  |
| Internship | 8 (5.1) | 37 (23.4) |  |
| Residency | 10 (6.3) | 27 (17.1) |  |
| Other | 15 (9.5) | 57 (36.1) |  |
| **Healthcare Service Delivery Setting** |  |  | 0.151 |
| Exclusively in private health settings | 9 (5.7) | 16 (10.1) |  |
| Exclusively in public health settings | 16 (10.1) | 66 (41.8) |  |
| In a combination of public health and private health settings | 9 (5.7) | 42 (26.6) |  |

|  | Agreed that using the phone to  consult with a patient will be easy for them | Disagreed that using the phone to  consult with a patient will be easy for them | P-Value |
| --- | --- | --- | --- |
| **Age** |  |  | 0.184 |
| 18-24 years | 4 (2.5) | 12 (7.6) |  |
| 25-48 years | 63 (39.9) | 79 (50.0) |  |
| **Geopolitical Zone** |  |  | 0.768 |
| North East | 3 (1.9) | 2 (1.3) |  |
| North-West | 3 (1.9) | 2 (1.3) |  |
| North Central | 3 (1.9) | 4 (2.5) |  |
| South-West | 9 (5.7) | 11 (7.0) |  |
| South-East | 19 (12.0) | 35 (22.2) |  |
| South-south | 30 (19.0) | 37 (23.4) |  |
| **Gender** |  |  | 0.14 |
| Female | 22 (13.9) | 41 (25.9) |  |
| Male | 45 (28.5) | 50 (31.6) |  |
| **Recent Professional Development Status** |  |  | 0.586 |
| Consultancy | 1 (0.6) | 3 (1.9) |  |
| Internship | 19 (12.0) | 26 (16.5) |  |
| Residency | 19 (12.0) | 18 (11.4) |  |
| Other | 28 (17.7) | 44 (27.8) |  |
| **Healthcare Service Delivery Setting** |  |  | 0.606 |
| Exclusively in private health settings | 13 (8.2) | 12 (7.6) |  |
| Exclusively in public health settings | 33 (20.9) | 49 (31.0) |  |
| In a combination of public health and private health settings | 21 (13.3) | 30 (19.0) |  |

|  | Agreed that video over the  internet will improve their teleconsultation experience and satisfaction | Disagreed that video over the  internet will improve their teleconsultation experience and satisfaction | P-Value |
| --- | --- | --- | --- |
| **Age** |  |  | 1 |
| 18-24 years | 10 (6.3) | 6 (3.8) |  |
| 25-48 years | 88 (55.7) | 54 (34.2) |  |
| **Geopolitical Zone** |  |  | 0.042 |
| North East | 2 (1.3) | 3 (1.9) |  |
| North-West | 4 (2.5) | 1 (0.6) |  |
| North Central | 7 (4.4) | 0 (0.0) |  |
| South-West | 16 (10.1) | 4 (2.5) |  |
| South-East | 34 (21.5) | 20 (12.7) |  |
| South-south | 35 (22.2) | 32 (20.3) |  |
| **Gender** |  |  | 1 |
| Female | 39 (24.7) | 24 (15.2) |  |
| Male | 59 (37.2) | 36 (22.8) |  |
| **Recent Professional Development Status** |  |  | <0.0001 |
| Consultancy | 3 (1.9) | 1 (0.6) |  |
| Internship | 35 (22.2) | 10 (6.3) |  |
| Residency | 29 (18.4) | 8 (5.1) |  |
| Other | 31 (19.6) | 41 (25.9) |  |
| **Healthcare Service Delivery Setting** |  |  | 0.128 |
| Exclusively in private health settings | 14 (8.9) | 11 (7.0) |  |
| Exclusively in public health settings | 57 (36.1) | 25 (15.8) |  |
| In a combination of public health and private health settings | 27 (17.1) | 24 (15.2) |  |

|  | Agreed that they would be as satisfied  talking to a patient over the phone as they would to a patient in-person in a consulting room a healthcare facility | Disagreed that they would be as satisfied  talking to a patient over the phone as they would to a patient in-person in a consulting room a healthcare facility | P-Value |
| --- | --- | --- | --- |
| **Age** |  |  | 0.361 |
| 18-24 years | 2 (1.3) | 14 (8.9) |  |
| 25-48 years | 37 (23.4) | 105 (66.5) |  |
| **Geopolitical Zone** |  |  | 0.515 |
| North East | 0 (0.0) | 5 (3.2) |  |
| North-West | 2 (1.3) | 3 (1.9) |  |
| North Central | 1 (0.6) | 6 (3.8) |  |
| South-West | 7 (4.4) | 13 (8.2) |  |
| South-East | 11 (7.0) | 43 (27.2) |  |
| South-south | 18 (11.4) | 49 (31.0) |  |
| **Gender** |  |  | 0.354 |
| Female | 13 (8.2) | 50 (31.6) |  |
| Male | 26 (16.5) | 69 (43.7) |  |
| **Recent Professional Development Status** |  |  | 0.697 |
| Consultancy | 1 (0.6) | 3 (1.9) |  |
| Internship | 9 (5.7) | 36 (22.8) |  |
| Residency | 8 (5.1) | 29 (18.4) |  |
| Other | 21 (13.3) | 51 (32.3) |  |
| **Healthcare Service Delivery Setting** |  |  | 0.557 |
| Exclusively in private health settings | 8 (5.1) | 17 (10.8) |  |
| Exclusively in public health settings | 18 (11.4) | 64 (40.5) |  |
| In a combination of public health and private health settings | 13 (8.2) | 38 (24.1) |  |

|  | Agreed that they prefer to  use teleconsultation services ONLY during pandemics | Disagreed that they prefer to  use teleconsultation services ONLY during pandemics | P-Value |
| --- | --- | --- | --- |
| **Age** |  |  | 0.189 |
| 18-24 years | 6 (3.8) | 10 (6.3) |  |
| 25-48 years | 80 (50.6) | 62 (39.2) |  |
| **Geopolitical Zone** |  |  | 0.635 |
| North East | 3 (1.9) | 2 (1.3) |  |
| North-West | 4 (2.5) | 1 (0.6) |  |
| North Central | 2 (1.3) | 5 (3.2) |  |
| South-West | 12 (7.6) | 8 (5.1) |  |
| South-East | 30 (19.0) | 24 (15.2) |  |
| South-south | 35 (22.2) | 32 (20.3) |  |
| **Gender** |  |  | 0.455 |
| Female | 32 (20.3) | 31 (19.6) |  |
| Male | 54 (34.2) | 41 (25.9) |  |
| **Recent Professional Development Status** |  |  | 0.225 |
| Consultancy | 2 (1.3) | 2 (1.3) |  |
| Internship | 27 (17.1) | 18 (11.4) |  |
| Residency | 24 (15.2) | 13 (8.2) |  |
| Other | 33 (20.9) | 39 (24.7) |  |
| **Healthcare Service Delivery Setting** |  |  | 0.285 |
| Exclusively in private health settings | 17 (10.8) | 8 (5.1) |  |
| Exclusively in public health settings | 41 (25.9) | 41 (25.9) |  |
| In a combination of public health and private health settings | 28 (17.7) | 23 (14.6) |  |

|  | Agreed that they would be  interested in being involved in a service offering medical consultations over the phone for patients | Disagreed that they would be  interested in being involved in a service offering medical consultations over the phone for patients | P-Value |
| --- | --- | --- | --- |
| **Age** |  |  | 0.246 |
| 18-24 years | 5 (3.2) | 11 (7.0) |  |
| 25-48 years | 66 (41.8) | 76 (48.1) |  |
| **Geopolitical Zone** |  |  | 0.783 |
| North East | 2 (1.3) | 3 (1.9) |  |
| North-West | 3 (1.9) | 2 (1.3) |  |
| North Central | 3 (1.9) | 4 (2.5) |  |
| South-West | 10 (6.3) | 10 (6.3) |  |
| South-East | 20 (12.7) | 34 (21.5) |  |
| South-south | 33 (20.9) | 34 (21.5) |  |
| **Gender** |  |  | 0.822 |
| Female | 29 (18.4) | 34 (21.5) |  |
| Male | 42 (26.6) | 53 (33.5) |  |
| **Recent Professional Development Status** |  |  | 0.294 |
| Consultancy | 1 (0.6) | 3 (1.9) |  |
| Internship | 25 (15.8) | 20 (12.7) |  |
| Residency | 17 (10.8) | 20 (12.7) |  |
| Other | 28 (17.7) | 44 (27.8) |  |
| **Healthcare Service Delivery Setting** |  |  | 0.061 |
| Exclusively in private health settings | 16 (10.1) | 9 (5.7) |  |
| Exclusively in public health settings | 37 (23.4) | 45 (28.5) |  |
| In a combination of public health and private health settings | 18 (11.4) | 33 (20.9) |  |
