# Supplementary material for: Healthcare provider-to-patient perspectives on the uptake of teleconsultation services in the Nigerian healthcare system during the COVID-19 pandemic era
Source: PLOS Glob Public Health. 2022 Feb 9;2(2):e0000189. doi: 10.1371/journal.pgph.0000189 (PMC10021919; doi:10.1371/journal.pgph.0000189)
Supplement: S2 Table — (DOCX) [file pgph.0000189.s005.docx]

**S2 Table: Results-Association between Healthcare Providers Demographic Characteristics and Perceptions on Benefits of Telemedicine Use**

|  | Agreed that Using the phone would be a useful (practical) and effective way for patients to receive healthcare service from a Doctor/Health professional during COVID-19 pandemic | Disagreed that Using the phone would be a useful (practical) and effective way for patients to receive healthcare service from a Doctor/Health professional during COVID-19 pandemic | P-Value |
| --- | --- | --- | --- |
| **Age** |  |  | 0.443 |
| 18-24 years | 7 (4.4) | 9 (5.7) |  |
| 25-48 years | 77 (48.7) | 65 (41.1) |  |
| **Geopolitical Zone** |  |  | 0.1 |
| North East | 4 (2.5) | 1 (0.6) |  |
| North-West | 3 (1.9) | 2 (1.3) |  |
| North Central | 3 (1.9) | 4 (2.5) |  |
| South-West | 16 (10.1) | 4 (2.5) |  |
| South-East | 27 (17.1) | 27 (17.1) |  |
| South-south | 31 (19.6) | 36 (22.8) |  |
| **Gender** |  |  | 0.872 |
| Female | 34 (21.5) | 29 (18.4) |  |
| Male | 50 (31.6) | 45 (28.5) |  |
| **Recent Professional Development Status** |  |  | 0.008 |
| Consultancy | 2 (1.3) | 2 (1.3) |  |
| Internship | 32 (20.3) | 13 (8.2) |  |
| Residency | 21 (13.3) | 16 (10.1) |  |
| Other | 29 (18.4) | 43 (27.2) |  |
| **Healthcare Service Delivery Setting** |  |  | 0.033 |
| Exclusively in private health settings | 17 (10.8) | 8 (5.1) |  |
| Exclusively in public health settings | 47 (29.7) | 35 (22.2) |  |
| In a combination of public health and private health settings | 20 (12.7) | 31 (19.6) |  |

|  | Agreed that using the phone would be an affordable way for patients to receive healthcare services during COVID-19 pandemic | Disagreed that using the phone would be an affordable way for patients to receive healthcare services during COVID-19 pandemic | P-Value |
| --- | --- | --- | --- |
| **Age** |  |  | 0.285 |
| 18-24 years | 12 (7.6) | 4 (2.5) |  |
| 25-48 years | 84 (53.2) | 58 (36.7) |  |
| **Geopolitical Zone** |  |  |  |
| North East | 3 (1.9) | 2 (1.3) | 0.226 |
| North-West | 4 (1.9) | 3 (1.3) |  |
| North Central | 5 (3.2) | 2 (1.3) |  |
| South-West | 17 (10.8) | 3 (1.3) |  |
| South-East | 31 (19.6) | 23 (14.6) |  |
| South-south | 37 (23.4) | 30 (19.0) |  |
| **Gender** |  |  | 0.62 |
| Female | 40 (25.3) | 23 (14.6) |  |
| Male | 56 (35.4) | 39 (24.7) |  |
| **Recent Professional Development Status** |  |  | 0.001 |
| Consultancy | 1 (0.6) | 3 (1.9) |  |
| Internship | 36 (22.8) | 9 (5.7) |  |
| Residency | 25 (15.8) | 12 (7.6) |  |
| Other | 34 (21.5) | 38 (24.1) |  |
| **Healthcare Service Delivery Setting** |  |  | 0.003 |
| Exclusively in private health settings | 20 (12.7) | 5 (3.2) |  |
| Exclusively in public health settings | 54 (34.2) | 28 (17.7) |  |
| In a combination of public health and private health settings | 22 (13.9) | 29 (18.4) |  |

|  | Agreed that using the phone would be a safe way for patients to receive healthcare services during COVID-19 pandemic | Disgreed that using the phone would be a safe way for patients to receive healthcare services during COVID-19 pandemic | P-Value |
| --- | --- | --- | --- |
| **Age** |  |  | 1 |
| 18-24 years | 10 (6.3) | 6 (3.8) |  |
| 25-48 years | 92 (58.2) | 50 (31.6) |  |
| **Geopolitical Zone** |  |  | 0.09 |
| North East | 4 (2.5) | 1 (0.6) |  |
| North-West | 2 (1.3) | 3 (1.9) |  |
| North Central | 6 (3.8) | 1 (0.6) |  |
| South-West | 17 (10.8) | 3 (1.9) |  |
| South-East | 36 (22.8) | 18 (11.4) |  |
| South-south | 37 (23.4) | 30 (19.0) |  |
| **Gender** |  |  | 0.866 |
| Female | 40 (25.3) | 23 (14.6) |  |
| Male | 62 (39.2) | 33 (20.9) |  |
| **Recent Professional Development Status** |  |  | 0.003 |
| Consultancy | 2 (1.3) | 2 (1.3) |  |
| Internship | 38 (24.1) | 7 (4.4) |  |
| Residency | 24 (15.2) | 13 (8.2) |  |
| Other | 38 (24.1) | 34 (21.5) |  |
| **Healthcare Service Delivery Setting** |  |  | 0.006 |
| Exclusively in private health settings | 19 (12.0) | 6 (3.8) |  |
| Exclusively in public health settings | 59 (37.3) | 23 (14.6) |  |
| In a combination of public health and private health settings | 24 (15.2) | 27 (17.1) |  |

|  | Agreed that delivering medical consultations and prescriptions to patients over the phone would be a convenient form of healthcare for patients | Disagreed that delivering consultations and prescriptions to patients over the phone would be a convenient form of healthcare for patients | P-Value |
| --- | --- | --- | --- |
| **Age** |  |  | 1 |
| 18-24 years | 6 (3.8) | 10 (6.3) |  |
| 25-48 years | 58 (36.7) | 84 (53.2) |  |
| **Geopolitical Zone** |  |  | 0.87 |
| North East | 2 (1.3) | 3 (1.9) |  |
| North-West | 1 (0.6) | 4 (2.5) |  |
| North Central | 3 (1.9) | 4 (2.5) |  |
| South-West | 10 (6.3) | 10 (6.3) |  |
| South-East | 20 (12.7) | 34 (21.5) |  |
| South-south | 28 (17.7) | 39 (24.7) |  |
| **Gender** |  |  | 0.62 |
| Female | 24 (15.2) | 39 (24.7) |  |
| Male | 40 (25.3) | 55 (34.8) |  |
| **Recent Professional Development Status** |  |  | 0.25 |
| Consultancy | 2 (1.3) | 2 (1.3) |  |
| Internship | 21 (13.3) | 24 (15.2) |  |
| Residency | 18 (11.4) | 19 (12.0) |  |
| Other | 23 (14.6) | 49 (31.0) |  |
| **Healthcare Service Delivery Setting** |  |  | 0.41 |
| Exclusively in private health settings | 12 (7.6) | 13 (8.2) |  |
| Exclusively in public health settings | 35 (54.7) | 47 (29.7) |  |
| In a combination of public health and private health settings | 17 (10.8) | 34 (21.5) |  |

|  | Agreed that delivering medical consultations and prescriptions to patients over the phone would save me time | Disagreed that delivering medical consultations and prescriptions to patients over the phone would save me time | P-Value |
| --- | --- | --- | --- |
| **Age** |  |  | 0.029 |
| 18-24 years | 14 (8.9) | 80 (50.6) |  |
| 25-48 years | 2 (1.3) | 62 (39.2) |  |
| **Geopolitical Zone** |  |  | 0.022 |
| North East | 3 (1.9) | 2 (1.3) |  |
| North-West | 5 (3.2) | 0 (0.0) |  |
| North Central | 7 (4.4) | 0 (0.0) |  |
| South-West | 12 (7.6) | 8 (5.1) |  |
| South-East | 35 (22.2) | 19 (12.0) |  |
| South-south | 32 (20.3) | 35 (22.2) |  |
| **Gender** |  |  | 0.741 |
| Female | 36 (22.8) | 27 (17.1) |  |
| Male | 58 (36.7) | 37 (23.4) |  |
| **Recent Professional Development Status** |  |  | 0.125 |
| Consultancy | 3 (1.9) | 1 (0.6) |  |
| Internship | 32 (20.3) | 13 (8.2) |  |
| Residency | 23 (14.6) | 14 (8.9) |  |
| Other | 36 (22.8) | 36 (22.8) |  |
| **Healthcare Service Delivery Setting** |  |  | 0.063 |
| Exclusively in private health settings | 20 (12.7) | 5 (3.2) |  |
| Exclusively in public health settings | 47 (29.7) | 35 (22.2) |  |
| In a combination of public health and private health settings | 27 (17.1) | 24 (15.2) |  |
