# Supplementary material for: Healthcare provider-to-patient perspectives on the uptake of teleconsultation services in the Nigerian healthcare system during the COVID-19 pandemic era
Source: PLOS Glob Public Health. 2022 Feb 9;2(2):e0000189. doi: 10.1371/journal.pgph.0000189 (PMC10021919; doi:10.1371/journal.pgph.0000189)
Supplement: S3 Table — (DOCX) [file pgph.0000189.s006.docx]

**S3 Table: Results-Association between Healthcare Consumers Demographic Characteristics and Perceptions on Telemedicine Use**

|  | Agreed that using the phone to  consult with a health provider will be easy for them | Disagreed that using the phone to  consult with a health provider will be easy for them | P-Value |
| --- | --- | --- | --- |
| **Age** |  |  | 0.005 |
| 18-24 years | 277 (20.1) | 54 (3.9) |  |
| 25-44 years | 784 (57.0) | 222 (16.1) |  |
| 45 years+ | 24 (1.7) | 14 (1.0) |  |
| **Geopolitical Zone** |  |  | <0.001 |
| North East | 9 (0.7) | 5 (0.4) |  |
| North-West | 19 (1.4) | 8 (0.6) |  |
| North Central | 70 (5.1) | 20 (1.5) |  |
| South-West | 319 (23.2) | 83 (6.0) |  |
| South-East | 472 (34.3) | 88 (6.4) |  |
| South-south | 196 (14.3) | 86 (6.3) |  |
| **Gender** |  |  | 0.39 |
| Female | 462(33.6) | 111(8.1) |  |
| Male | 621 (45.2) | 178 (12.9) |  |
| **Highest Education Level** |  |  | <0.001 |
| Bachelors | 539 (39.2) | 177 (12.9) |  |
| Secondary | 262 (19.1) | 29 (2.1) |  |
| Masters | 221 (16.1) | 62 (4.5) |  |
| Doctorate | 31 (2.3) | 10 (0.7) |  |
| Others | 28 (2.0) | 7 (0.5) |  |
| No Formal Education | 4 (0.3) | 5 (0.4) |  |
| **Academic Background** |  |  | 0.014 |
| Non-Scientific/Non-medical | 560 (40.7) | 126 (9.2) |  |
| Scientific/Medical | 525 (38.2) | 164 (11.9) |  |

|  | Agreed that video over the  internet will improve their teleconsultation experience and satisfaction | Disagreed that video over the  internet will improve their teleconsultation experience and satisfaction | P-Value |
| --- | --- | --- | --- |
| **Age** |  |  | 0.584 |
| 18-24 years | 279 (20.3) | 52 (3.8) |  |
| 25-44 years | 823 (59.9) | 183 (13.3) |  |
| 45 years+ | 31 (2.3) | 7 (0.5) |  |
| **Geopolitical Zone** |  |  | <0.001 |
| North East | 5 (0.4) | 9 (0.7) |  |
| North-West | 21 (1.5) | 6 (0.4) |  |
| North Central | 78 (5.7) | 12 (0.9) |  |
| South-West | 349 (25.4) | 53 (3.9) |  |
| South-East | 489 (35.9) | 71 (5.2) |  |
| South-south | 191 (13.9) | 91 (6.6) |  |
| **Gender** |  |  | 0.88 |
| Female | 473 (34.4) | 100 (7.3) |  |
| Male | 657 (47.8) | 142 (10.3) |  |
| **Highest Education Level** |  |  | <0.001 |
| Bachelors | 567 (41.2) | 149 (10.8) |  |
| Secondary | 268 (19.5) | 23 (1.7) |  |
| Masters | 231 (16.8) | 52 (3.8) |  |
| Doctorate | 32 (2.3) | 9 (0.7) |  |
| Others | 31 (2.3) | 4 (0.3) |  |
| No Formal Education | 4 (0.3) | 5 (0.4) |  |
| **Academic Background** |  |  | 0.077 |
| Non-Scientific/Non-medical | 578 (42.0) | 108 (7.9) |  |
| Scientific/Medical | 555 (40.4) | 134 (9.7) |  |

|  | Agreed that they would be as satisfied  talking to a health provider over the phone as they would to a health provider in-person in a consulting room a healthcare facility | Disagreed that they would be as satisfied  talking to a health provider over the phone as they would to a health provider in-person in a consulting room a healthcare facility | P-Value |
| --- | --- | --- | --- |
| **Age** |  |  | <0.0001 |
| 18-24 years | 261 (19.0) | 70 (5.1) |  |
| 25-44 years | 678 (49.3) | 328 (23.9) |  |
| 45 years+ | 18 (1.3) | 20 (1.5) |  |
| **Geopolitical Zone** |  |  | <0.01 |
| North East | 9 (0.7) | 5 (0.4) |  |
| North-West | 15 (1.1) | 12 (0.9) |  |
| North Central | 51 (3.7) | 39 (2.8) |  |
| South-West | 275 (20.0) | 127 (9.2) |  |
| South-East | 420 (30.5) | 140 (10.2) |  |
| South-south |  |  |  |
| **Gender** |  |  | 0.72 |
| Female | 400 (29.1) | 173 (12.6) |  |
| Male | 554 (40.3) | 245 (17.8) |  |
| **Highest Education Level** |  |  | <0.0001 |
| Bachelors | 473 (34.4) | 243 (17.7) |  |
| Secondary | 257 (18.7) | 34 (2.5) |  |
| Masters | 181 (13.2) | 102 (7.4) |  |
| Doctorate | 20 (1.5) | 21 (1.5) |  |
| Others | 22 (13) | 13 (0.9) |  |
| No Formal Education | 4 (0.3) | 5 (0.4) |  |
| **Academic Background** |  |  | <0.0001 |
| Non-Scientific/Non-medical | 510 (37.1) | 176 (12.8) |  |
| Scientific/Medical | 447 (32.5) | 242 (17.6) |  |

|  | Agreed that they prefer to  use teleconsultation services ONLY during pandemics | Disagreed that they prefer to  use teleconsultation services ONLY during pandemics | P-Value |
| --- | --- | --- | --- |
| **Age** |  |  | <0.0001 |
| 18-24 years | 235 (17.1) | 96 (7.0) |  |
| 25-44 years | 534 (38.8) | 472 (34.3) |  |
| 45 years+ | 22 (1.6) | 16 (1.2) |  |
| **Geopolitical Zone** |  |  | <0.001 |
| North East | 7 (0.5) | 7 (0.5) |  |
| North-West | 14 (1.0) | 13 (0.9) |  |
| North Central | 31 (2.3) | 59 (4.3) |  |
| South-West | 186 (13.5) | 216 (15.7) |  |
| South-East | 385 (28.0) | 175 (12.7) |  |
| South-south | 168 (12.2) | 114 (8.3) |  |
| **Gender** |  |  | 0.14 |
| Female | 348 (25.3) | 225 (16.4) |  |
| Male | 442 (32.1) | 357 (26.0) |  |
| **Highest Education Level** |  |  | <0.0001 |
| Bachelors | 353 (25.7) | 363 (26.4) |  |
| Secondary | 256 (18.6) | 35 (2.5) |  |
| Masters | 135 (9.8) | 148 (10.8) |  |
| Doctorate | 18 (1.3) | 23 (1.7) |  |
| Others | 25 (1.8) | 10 (0.7) |  |
| No Formal Education | 4 (0.3) | 5 (0.4) |  |
| **Academic Background** |  |  | <0.0001 |
| Non-Scientific/Non-medical | 449 (32.7) | 237 (17.2) |  |
| Scientific/Medical | 342 (24.9) | 347 (25.2) |  |

|  | Agreed that they would be  interested in being involved in a service offering medical consultations over the phone for patients | Disagreed that they would be  interested in being involved in a service offering medical consultations over the phone for patients | P-Value |
| --- | --- | --- | --- |
| **Age** |  |  | <0.0001 |
| 18-24 years | 271 (19.7) | 60 (4.4) |  |
| 25-44 years | 702 (51.1) | 304 (22.1) |  |
| 45 years+ | 24 (1.7) | 14 (1.0) |  |
| **Geopolitical Zone** |  |  | <0.0001 |
| North East | 8 (0.6) | 6 (0.4) |  |
| North-West | 16 (1.2) | 11 (0.8) |  |
| North Central | 65 (4.7) | 25 (1.8) |  |
| South-West | 297 (21.6) | 105 (7.6) |  |
| South-East | 447 (32.5) | 113 (8.2) |  |
| South-south | 164 (11.9) | 118 (8.6) |  |
| **Gender** |  |  | 0.414 |
| Female | 427 (31.1) | 146 (10.6) |  |
| Male | 568 (41.3) | 231 (16.8) |  |
| **Highest Education Level** |  |  | <0.0001 |
| Bachelors | 495 (36.0) | 221 (16.1) |  |
| Secondary | 263 (19.1) | 28 (2.0) |  |
| Masters | 190(13.8) | 93 (6.8) |  |
| Doctorate | 23 (1.7) | 18 (1.3) |  |
| Others | 22 (1.6) | 13 (0.9) |  |
| No Formal Education | 4 (0.3) | 5 (0.4) |  |
| **Academic Background** |  |  | 0.005 |
| Non-Scientific/Non-medical | 521 (37.9) | 165 (12.0) |  |
| Scientific/Medical | 476 (34.6) | 213 (15.5) |  |
