# Supplementary material for: Healthcare provider-to-patient perspectives on the uptake of teleconsultation services in the Nigerian healthcare system during the COVID-19 pandemic era
Source: PLOS Glob Public Health. 2022 Feb 9;2(2):e0000189. doi: 10.1371/journal.pgph.0000189 (PMC10021919; doi:10.1371/journal.pgph.0000189)
Supplement: S4 Table — (DOCX) [file pgph.0000189.s007.docx]

**S4 Table: Results-Association between Healthcare Consumers Demographic Characteristics and Perceptions on Benefits of Telemedicine Use**

|  | Agreed that Using the phone would be a useful (practical) and effective way for patients to receive healthcare service from a Doctor/Health professional during COVID-19 pandemic | Disagreed that Using the phone would be a useful (practical) and effective way for patients to receive healthcare service from a Doctor/Health professional during COVID-19 pandemic | P-Value |
| --- | --- | --- | --- |
| **Age** |  |  | 0.018 |
| 18-24 years | 287 (20.9) | 44 (3.2) |  |
| 25-44 years | 821 (59.7) | 185 (13.5) |  |
| 45 years+ | 27 (2.0) | 11 (0.8) |  |
| **Geopolitical Zone** |  |  | <0.0001 |
| North East | 11 (0.8) | 3 (0.2) |  |
| North-West | 23 (1.7) | 4 (0.3) |  |
| North Central | 76 (5.5) | 14 (1.0) |  |
| South-West | 349 (25.4) | 53 (3.9) |  |
| South-East | 485 (35.3) | 75 (5.5) |  |
| South-south | 191 (13.9) | 91 (6.6) |  |
| **Gender** |  |  | 0.421 |
| Female | 483 (35.1) | 90 (6.5) |  |
| Male | 649 (47.2) | 150 (10.9) |  |
| **Highest Education Level** |  |  | <0.0001 |
| Bachelors | 583 (42.4) | 133 (9.7) |  |
| Secondary | 266 (19.3) | 25 (1.8) |  |
| Masters | 224 (16.3) | 59 (4.3) |  |
| Doctorate | 30 (2.2) | 11 (0.8) |  |
| Others | 28 (2.0) | 7 (0.5) |  |
| No Formal Education | 4 (0.3) | 5 (0.4) |  |
| **Academic Background** |  |  | 0.177 |
| Non-Scientific/Non-medical | 576 (41.9) | 110 (8.0) |  |
| Scientific/Medical | 559 (40.7) | 130 (9.5) |  |

|  | Agreed that using the phone would be an affordable way for patients to receive healthcare services during COVID-19 pandemic | Disagreed that using the phone would be an affordable way for patients to receive healthcare services during COVID-19 pandemic | P-Value |
| --- | --- | --- | --- |
| **Age** |  |  | 0.05 |
| 18-24 years | 288 (20.9) | 43 (3.1) |  |
| 25-44 years | 816 (59.3) | 190 (13.8) |  |
| 45 years+ | 31 (2.3) | 7 (0.5) |  |
| **Geopolitical Zone** |  |  | <0.0001 |
| North East | 11 (0.8) | 3 (0.2) |  |
| North-West | 22 (1.6) | 5 (0.4) |  |
| North Central | 79 (5.7) | 11 (0.8) |  |
| South-West | 347 (25.2) | 55 (4.0) |  |
| South-East | 480 (34.9) | 80 (5.8) |  |
| South-south |  |  |  |
| **Gender** |  |  | 0.496 |
| Female | 479 (34.8) | 94 (6.8) |  |
| Male | 654 (47.6) | 145 (10.5) |  |
| **Highest Education Level** |  |  | <0.0001 |
| Bachelors | 580 (42.2) | 136 (9.9) |  |
| Secondary | 266 (19.3) | 25 (1.8) |  |
| Masters | 224 (16.3) | 59 (4.3) |  |
| Doctorate | 33 (2.4) | 8 (0.6) |  |
| Others | 28 (2.0) | 7 (0.5) |  |
| No Formal Education | 4 (0.3) | 5 (0.4) |  |
| **Academic Background** |  |  | 0.076 |
| Non-Scientific/Non-medical | 579 (42.1) | 107 (7.8) |  |
| Scientific/Medical | 556 (40.4) | 133 (9.7) |  |

|  | Agreed that using the phone would be a safe way for patients to receive healthcare services during COVID-19 pandemic | Disagreed that using the phone would be a safe way for patients to receive healthcare services during COVID-19 pandemic | P-Value |
| --- | --- | --- | --- |
| **Age** |  |  | 0.039 |
| 18-24 years | 290 (21.1) | 41 (3.0) |  |
| 25-44 years | 826 (60.1) | 180 (13.1) |  |
| 45 years+ | 34 (2.5) | 4 (0.3) |  |
| **Geopolitical Zone** |  |  | <0.0001 |
| North East | 9 (0.7) | 5 (0.4) |  |
| North-West | 24 (1.7) | 3 (0.2) |  |
| North Central | 81 (5.9) | 9 (0.7) |  |
| South-West | 356 (31.0) | 46 (3.3) |  |
| South-East | 490 (35.6) | 70 (5.1) |  |
| South-south | 190 (13.8) | 92 (6.7) |  |
| **Gender** |  |  | 0.104 |
| Female | 495 (36.0) | 78 (5.7) |  |
| Male | 652 (47.4) | 147 (10.7) |  |
| **Highest Education Level** |  |  | <0.0001 |
| Bachelors | 574 (41.7) | 142 (10.3) |  |
| Secondary | 267 (19.4) | 24 (1.7) |  |
| Masters | 245 (17.8) | 38 (2.8) |  |
| Doctorate | 29 (2.1) | 12 (0.9) |  |
| Others | 31 (2.3) | 4 (0.3) |  |
| No Formal Education | 4 (0.3) | 5 (0.4) |  |
| **Academic Background** |  |  | 0.029 |
| Non-Scientific/Non-medical | 589 (42.8) | 97 (7.1) |  |
| Scientific/Medical | 561 (40.8) | 128 (9.3) |  |

|  | Agreed that Receiving medical consultations and prescriptions from a Doctor/Health professional over the phone would be a convenient form of healthcare for patients | Disagreed that Receiving medical consultations and prescriptions from a Doctor/Health professional over the phone would be a convenient form of healthcare for patients | P-Value |
| --- | --- | --- | --- |
| **Age** |  |  | <0.0001 |
| 18-24 years | 266 (19.3) | 65 (4.7) |  |
| 25-44 years | 662 (48.1) | 344 (25.0) |  |
| 45 years+ | 26 (1.9) | 12 (0.9) |  |
| **Geopolitical Zone** |  |  | <0.0001 |
| North East | 6 (0.4) | 8 (0.6) |  |
| North-West | 17 (1.2) | 10 (0.7) |  |
| North Central | 67 (4.9) | 23 (1.7) |  |
| South-West | 289 (21.0) | 113 (8.2) |  |
| South-East | 419 (30.5) | 141 (10.3) |  |
| South-south | 156 (11.3) | 126 (9.2) |  |
| **Gender** |  |  | 0.452 |
| Female | 409 (29.7) | 164 (11.9) |  |
| Male | 543 (39.5) | 256 (18.6) |  |
| **Highest Education Level** |  |  | <0.0001 |
| Bachelors | 461 (33.5) | 255 (18.5) |  |
| Secondary | 253 (18.4) | 38 (2.8) |  |
| Masters | 190 (13.8) | 93 (6.8) |  |
| Doctorate | 27 (2.0) | 14 (1.0) |  |
| Others | 21 (1.5) | 14 (1.0) |  |
| No Formal Education | 2 (0.1) | 7 (0.5) |  |
| **Academic Background** |  |  | <0.0001 |
| Non-Scientific/Non-medical | 512 (37.2) | 174 (12.7) |  |
| Scientific/Medical | 442 (32.1) | 247 (18.0) |  |

|  | Agreed that receiving medical consultations and prescriptions from a Doctor/Health professional over the phone would save me time | Disagreed that receiving medical consultations and prescriptions from a Doctor/Health professional over the phone would save me time | P-Value |
| --- | --- | --- | --- |
| **Age** |  |  | 0.077 |
| 18-24 years | 285 (20.7) | 46 (3.3) |  |
| 25-44 years | 812 (59.1) | 194 (14.1) |  |
| 45 years+ | 30 (2.2) | 8 (0.6) |  |
| **Geopolitical Zone** |  |  | <0.0001 |
| North East | 6 (0.4) | 8 (0.6) |  |
| North-West | 17 (1.2) | 10 (0.7) |  |
| North Central | 75 (5.5) | 23 (1.7) |  |
| South-West | 289 (21.0) | 113 (8.2) |  |
| South-East | 419 (30.5) | 141 (10.3) |  |
| South-south | 156 (11.3) | 126 (9.2) |  |
| **Gender** |  |  | 0.222 |
| Female | 483 (35.1) | 90 (6.5) |  |
| Male | 641 (46.6) | 158 (11.5) |  |
| **Highest Education Level** |  |  | <0.0001 |
| Bachelors | 571 (41.5) | 145 (10.5) |  |
| Secondary | 264 (19.2) | 27 (2.0) |  |
| Masters | 229 (16.7) | 54 (3.9) |  |
| Doctorate | 32 (2.3) | 9 (0.7) |  |
| Others | 27 (2.0) | 8 (0.6) |  |
| No Formal Education | 4 (0.3) | 5 (0.4) |  |
| **Academic Background** |  |  | 0.233 |
| Non-Scientific/Non-medical | 571 (41.5) | 115 (8.4) |  |
| Scientific/Medical | 556 (40.4) | 133 (9.7) |  |
