# Supplementary material for: Healthcare provider-to-patient perspectives on the uptake of teleconsultation services in the Nigerian healthcare system during the COVID-19 pandemic era
Source: PLOS Glob Public Health. 2022 Feb 9;2(2):e0000189. doi: 10.1371/journal.pgph.0000189 (PMC10021919; doi:10.1371/journal.pgph.0000189)
Supplement: Attachment — Submitted filename: Response to Reviewers.pdf [file pgph.0000189.s008.pdf]

Institut de Recerca de la Sida. IrsiCaixa.
Hospital Universitari Germans Trias i Pujol
Ctra. de Canyet s/n. 2a Planta Maternal. 08916 Badalona. Barcelona
F +34 934 653 968 / www.irsicaixa.es

December 20, 2021

PLOS GLOBAL PUBLIC HEALTH
Editorial Office

Dear Editor,

I would like to submit, on behalf of all co-authors, the rebuttal to the reviewers' comments for the manuscript entitled "Healthcare provider-to-Patient perspectives on the uptake of Teleconsultation services in the Nigerian healthcare system in the COVID-19 pandemic era".

We sincerely appreciate the reviewers for assessing our study and providing us with these feedbacks. The responses to the reviewers' comments are highlighted below in blue texts.

Reviewer #1:

The authors have done a good job in this manuscript. The data analysis and presentation is excellent, and also the conclusion is well supported with the data in the results. However, there are some minor corrections required in the general text as follows:
♣ Lines 152-153: delete "Healthcare providers" and use only its abbreviation because it was already abbreviated in Lines 112-113. This has been corrected

♣ Lines 179-180: delete "healthcare providers" (line-179) and "healthcare consumers" (line-180) and use only their abbreviations. This has been corrected

♣ Line 182: long form of UBTH is important to be inserted followed by UBTH in brackets, i.e., University of Benin Teaching Hospital (UBTH). Then, in subsequent areas where UBTH is written in long form, you can replace it with its abbreviation. This has been corrected

♣ There is a need for consistency in the use of abbreviation for the Coronavirus disease of 2019. In most lines in the Introduction part, authors have used "Covid-19" but in other manuscript sections (including in the abstract), they have used COVID-19. For example, in Lines 185, 241, 279, 282, 286, etc., they used "COVID-19". This has been corrected

♣ Lines 380-381: delete "healthcare providers" (line-380) and "healthcare consumers" (line-381) and use only their abbreviations. This has been corrected

♣ Line 414: “responses from healthcare providers and healthcare consumers,”, the authors need to use the abbreviations for HCP and HCC instead of their long-form. This has been corrected

♣ Line 417: “telemedicine uptake, previous studies have reported that healthcare providers” – use HCP instead of its long form. This has been corrected

♣ Line 420: “telemedicine for healthcare consumers is a critical factor in telemedicine uptake” – use HCC instead of its long form. This has been corrected

♣ Line 434: “Nigerian healthcare providers and the general public. This information is critical” – replace “healthcare providers” with HCP after the word “Nigerian”. This has been corrected

♣ Line 442: “be generalizable to the healthcare providers and the public in Nigeria. However,” – replace “healthcare provider” with HCP. This has been corrected

♣ Lines 450-452: “using an online survey. Notwithstanding the limitations, the results of this study provide information providers' and individuals' perceptions on the use of telemedicine due to the coronavirus pandemic.” I SUGGEST THAT THE AUTHORS NEED TO REPHRASE IT TO READ AS FOLLOWS: ““using an online survey. Notwithstanding the limitations, the results of this study provide information REGARDING providers' and individuals' perceptions on the use of telemedicine due to the (((DELETE THIS coronavirus and insert))) COVID-19 pandemic.” This has been corrected

♣ Lines 464-465: “barriers. In this study, most participants, healthcare providers, and healthcare consumers expressed positive…….” – replace “healthcare providers, and healthcare consumers” with “HCP and HCC”. This has been corrected

Reviewer #2:

The author of the paper has done good work. The issues identified are:-
1. Study population is not clear. How many health care providers were sampled and how they identified, also to the healthcare consumers. how was their conduct obtained e.g. mobile number or how did participants both HCP and

The description of the study participants are presented in the methods (pg 5-6; Lines 160-175). The sample size for the HCC was estimated using the OpenEpi application where we determined the required sample for HCC to be = 1034 respondents. In addition, we added a 30% contingency to this estimate to arrive at 1345 respondents (HCC). Regarding the HCP, we assumed an HCP:HCC ratio of 1:10 to arrive at 135 respondents (HCP). Recruitment of study participants was made via social media such as WhatsApp, Facebook (meta), Twitter and LinkedIn (Lines 170-172).

3. How easy was healthcare consumers getting health providers conducts like phones and how was the consumers able to authenticate the numbers. Consumers recruited to the social media used to even deliver google forms used?.

We did not explore this narrative, and so could not determine the modality or authenticity of HCP-HCC contact via phone. Moreover, the responses were completely anonymous. One of the limitations of this study which we have highlighted in the manuscript is the selection bias inherent with convenience sampling technique used in this study (Lines 432-435). ¨This means that the survey was limited to individuals who have smartphone devices and were technologically savvy to complete online surveys.

What was the response rate? Was stratification of the samples We obtained 100% and 98.7% response rates for HCP and HCC respectively. No stratification was done for this study.

2. Only one question for healthcare consumers as per the chart.

The responses for both HCP and HCC were summarized in two figures each with similar questions – Figs 1 and 2 (HCP), and Figs 3 and 4 (HCC).

Thank You.

Sincerely,

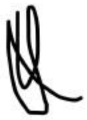

Ifeanyi Jude Ezeonwumelu
Virus-Host Interactions Team (ViHIT)
IrsiCaixa - AIDS Research Institute,
Health Research Institute Germans Trias I Pujol (IGTP),
Hospital Germans Trias i Pujol, Badalona;
iezeonwumelu@irsicaixa.es
T. (+34) 93 465 63 74 - Ext. 229

Universitat Autònoma de Barcelona,
Barcelona, Spain.
